# Effects of Partial Replacement of NaCl with KCl on Protein Properties and Quality Attributes of Lightly Salted Tilapias Fillets

**DOI:** 10.3390/foods12061184

**Published:** 2023-03-11

**Authors:** Lunan Jing, Jingqi Xue, Xin Jiang, Naiyong Xiao, Hao Pan, Jiarou Li, Dajun Wang, Qingqing Jiang, Wenzheng Shi

**Affiliations:** 1 College of Food Science and Technology, Shanghai Ocean University, Shanghai 201306, China; 2 Yantai Haiyu Foodstuffs Co., Ltd. (Yantai), Yantai 264000, China; 3 National R&D Branch Center for Freshwater Aquatic Products Processing Technology (Shanghai), Shanghai 201306, China

**Keywords:** lightly salted, KCl replacements, protein structural stability, sensory quality

## Abstract

The evolution of quality attributes and their association with the protein properties of lightly tilapias fillets salted with different replacement proportions of NaCl with KCl (0%, 10%, 30%, 50%, 70%, 100%) at the same ionic strength were investigated. KCl replacements using optimal substitution (50% of KCl) contributed to maintaining desired quality properties. Further, KCl replacement (about 50~70% of KCl) led to the insolubilization and weakened stability of myofibrillar proteins, represented by the unfolding of the myofibrillar protein, increased surface hydrophilic points, and strengthened internal protein-protein interaction, resulting in the structurally reinforced hardness and lower water-holding capacity. Excessive replacement (more than 70% of KCl) showed apparent deterioration in taste quality, coloration, and hardness received by sensory sensation caused by immoderate hydrolysis and aggravated oxidation of the myofibrillar protein. In this sense, insights into KCl replacements on protein properties might be a positive approach to improving quality attributes of lightly salted tilapias fillets.

## 1. Introduction

Salting plays a crucial role both in prolonging the shelf-life and contributing to the intense salty flavor of perishable fish and fish products [1]. Sodium chloride (NaCl), as an essential ingredient, can make aquatic products available, regardless of regional distance or seasonal mismatch, and can confer a unique flavor [2]. Tilapias (*Oreochromis niloticus*), with its advantages of wide distribution and rapid reproduction, have been the main aquaculture product processed as salted fillets in China. Due to its superior edible value of richness in protein, essential amino and polyunsaturated fatty acids, tilapias are also associated with health benefits. However, particularly in tender, moist tissue and tissue that is intrinsically perishable, tilapias are highly sensitive to several chemical and biochemical changes, which directly affect the quality of food. Furthermore, no attempt has been made for the fish matrix, let alone inconsistent conclusions. More importantly, the fact has been proven in recent years that a diet of high dietary sodium salt intake is a significant cause of hypertension and cardiovascular disease [3]. Therefore, a high-sodium diet was, is, and will continue to be one of the vital factors restricting the development of the salted fish and seafood industry [4]. The main approaches, currently, are focused on the reduction of the NaCl added or substitution with other salts such as KCl. Due to the similarity between KCl and NaCl in molecular composition, saltiness, and antibacterial activity [5], not to mention its ability to lower blood pressure [6], KCl is more commonly referred to as an alternative substitution [7]. However, sodium reduction may decrease the sensory qualities and extraction of the myofibrillar proteins (actin and myosin), which are essential for the moisture content and water-−holding capacities [8]. KCl substitution alone can be limited to texture deterioration and the perception of undesirable sensory attributes, negatively influencing consumers’ acceptance of the product [5]. In this sense, we should understand the effects precisely to overcome the quality deterioration of low-sodium salt in tilapia fillets.

Quality attributes and physicochemical properties of tissue mainly depend on the myofibrillar and actomyosin in muscle fibers [9]. Protein structural characteristics as an important factor directly affect a series of changes in functional groups of the protein [10], thus leading to sensory characteristics of fish and fish products [11]. As in KCl and NaCl co-−salting treatment, Na^+^ with a higher hydration radius more effectively stabilized the system than K^+^ at the same valency under high salt concentration [7]. According to the stabilization mechanism of the hydrophilic nature of the interface [12], more proteins absorbed by K^+^ resulted in weaker hydration stability [13]. It seems reasonable to speculate that higher levels of KCl tend to change protein conformation, which could affect protein conformation and water loss. Similarly, K^+^ with a greater ion radius but low charge density leads to a weaker electrostatic effect [14]. From this perspective, further intensive study of the synergistic effect of KCl and NaCl on quality attributes and protein properties plays an essential role in the production practice of the low-sodium salt industry.

Inconsistencies in findings within and across individual studies raise concerns about whether KCl and NaCl co-salting affects protein properties and the sensory quality of fish fillets, and the mechanism involved. While more emphasis was placed on the taste and texture deterioration of fish and fish products by KCl substitution, the effect on sensory quality and protein properties by synergistic salting of KCl and NaCl was ignored. The purpose of this study is to compare and explore changes in physicochemical characteristics, sensory quality, proteolysis, and myofibrillar protein structure, and to elucidate the relationship between protein conformation and quality attributes of tilapia fillets as affected by the partial substitution of NaCl with KCl to arrive at a conclusion.

## 2. Materials and Methods

### 2.1. Sample Preparation

Fresh tilapia (weight: 105.0 ± 5.0 g, length: 14.5 ± 0.8 cm) was selected as the raw material, purchased from a local aquatic store (Shanghai, China). Raw tilapia guts were trimmed away, cleaned, chilled, and then the dorsal flesh was sliced into 5 cm × 3 cm × 1 cm samples, unless otherwise specified. The prepared samples were salted in 6 mol∙L^−1^ pickle brine with different proportions of KCl replacing NaCl (10%, 30%, 50%, 70%, 100%) as KCl-treated groups. Based on the results of the pre-test, samples were salted at 4.0 ± 0.5 °C, and immersed in a pre-cooling solution with a slice to solution ratio of 1:3 (w:w) for 4 h to obtain the best sensory quality. Samples salted in 6 mol∙L^−1^ NaCl solution were used as a control (not replaced). Protein properties were evaluated after salting; the remaining materials were minced and stored at −80 °C before analyses of physicochemical properties.

### 2.2. Measurement of Moisture Content

The moisture content was expressed as a mass ratio after drying to that before drying at 105 °C for 12 h.

(1) $$\mathrm{Moisture}\;\mathrm{content}\;\;\left(\%\right)=\left(m_{1}-m_{2}\right)/m_{1}\times 100$$

where $m_{1}$ and $m_{2}$ indicate the weight after salting and after drying at 105 °C for 12 h, respectively.

### 2.3. Measurement of Yield

The yield was expressed as a mass ratio of the sample after salting to that before salting.

(2) $$\mathrm{Yield}\;\left(\%\right)=m_{1}/m_{0}\times 100$$

where $m_{0}$ and $m_{1}$ indicate the weight before salting and after salting, respectively.

### 2.4. Measurement of the Cooking Loss and the Centrifugal Water Loss

The cooking loss rate was measured based on the method proposed by Song et al. [15] with minor modifications. Samples (5.0 g) were placed into every corresponding centrifuge tube, accompanied by a water bath at 80.0 ± 1.0 °C for 10 min. Then, samples were cooled at ambient temperature and the moisture was gently wiped off the surface. The centrifugal water loss rate was determined by the procedure [14]. The sample was wrapped with absorbent paper and centrifuged at 800× *g* for 20 min at 4 °C. The cooking loss, the centrifugal water loss, and water holding capacity (WHC) were calculated as follows:

(3) $$\mathrm{Centrifugal}\;\mathrm{loss}\;\left(\%\right)=\left(m_{1}-m_{3}\right)/m_{1}\times 100$$

(4) $$\mathrm{Cooking}\;\mathrm{loss}\;\left(\%\right)=\left(m_{1}-m_{4}\right)/m_{1}\times 100$$

where $m_{1}$, $m_{3}$, and $m_{4}$ indicate the weight after salting, that is, before centrifuging or cooking, after centrifuging at 800× *g* for 20 min at 4 °C, and after cooking for 10 min at −80 °C, respectively.

### 2.5. Measurement of pH

In total, 2.00 g of minced samples were scattered in 18 mL of pre-cooling distilled water. The homogenate was centrifuged for 10 min at 10,000 r∙min^−1^. The pH of the supernatant was measured using a digital pH meter (FE20, Mettler Toledo Scientific Instrument Co., Ltd., Shanghai, China).

### 2.6. Texture Profile Analysis (TPA)

Texture properties of tilapia fillets of the size 2 cm × 2 cm × 1 cm with different KCl substitution proportions were subject to TA. XTplus texture analyzer (SMS TA.XT Plus, Stable Micro Systems Co., Ltd., Godalming, UK) using a P/50 flat-bottomed cylindrical probe evaluated texture in terms of hardness, springiness, cohesiveness, and chewiness. Text parameters were as follows: The pre-test velocity was 3.00 mm∙s^−1^, the test velocity was 1.00 mm∙s^−1^, and the return velocity was 1.00 mm∙s^−1^. In two compression programs, the trigger force was 5 g, with a compression degree of 50%, and the interval compression processing time was 5 s. For each group of samples, 12 parallels were selected for TPA.

### 2.7. Color Analysis

The color analysis of salted tilapia fillets was performed using a colorimeter (CR-20, Minolta Scientific Instrument Co., LTD, Osaka, Japan). This was followed by the CIE color system calibrated with the built-in whiteboard in the chromaticity meter; chromaticity was identified and recorded by attaching the sample to the transparent hole completely. The L* value represented lightness, a* value represented redness and greenness, whereas the b* value represented yellowness and blueness.

### 2.8. Sensory Evaluation

The samples prepared with KCl and NaCl in different proportions were steamed with the boiled water for 8 min and cooled to 40 °C before they were carried out to assess the synergistic effect of KCl and NaCl in different proportions on sensory properties. Five aspects (color intensity, texture intensity, aroma intensity, taste intensity, and overall acceptability) were subject to quantitative descriptive analyses (QDA) and scored by ten professionally trained sensory evaluators in the sensory evaluation laboratory of Shanghai Ocean University. The intensity of every attribute was expressed on an unstructured scale from 0 (the sensation not perceived) to 10 (maximum sensation perceived). The final scores were averaged across all panelists.

### 2.9. Preparation of Myofibrillar Proteins

In total, 20 mL 20 mM phosphate buffer A (containing 0.1 M NaCl, 1 mM EDTA, pH 7.0) was added and homogenized with 1.0 g minced samples. The homogenate was centrifuged at 10,000 r∙min^−1^ at 4 °C for 10 min. The above operation was performed twice more and retained the precipitation. Then, the homogenate of the precipitate and 25 mL 20 mM phosphate buffer B (containing 0.6 M NaCl, pH 7.0) were placed in a refrigerator for 2 h. Insoluble connective tissue was removed with two layers of gauze, and the filtrate was myofibril solution. The protein concentration was determined by the biuret method using BSA as standard [16], and the concentration was diluted to 4.0 mg∙mL^−1^ by buffer B.

### 2.10. Measurement of Proteolysis Index (PI)

To clarify the changes in proteolysis of KCl and NaCl co-salted tilapias fillet in different proportions, both the total nitrogen (TN) and non-protein nitrogen (NPN) were subjected to the Kjeldahl procedure of nitrogen determination analysis following the method [17]. As for total TN level, 0.2 g minced samples, after digestion with 12 mL 98% sulfuric acid at 420 °C for 1.5 h, were determined by automatic Kjeldahl apparatus. As for NPN level, 5 g minced samples were homogenized with citric acid buffer and placed for 2 h at 4 °C. Then, they were centrifuged at 10,000 r∙min^−1^ for 15 min. The filtered supernatant was mixed in 20 mL 10% trichloroacetic acid and placed overnight at 4 °C. Then, it was centrifuged at 10,000 r∙min^−1^ for 5 min; the filtered supernatant was digested and assessed using the same method as TN determination. The proteolysis index (PI) is expressed as the ratio of NPN to TN.

(5) PI%=NPNTN×100

### 2.11. SDS-PAGE

To clarify the changes in proteins of salted tilapias fillet at different proportions of KCl instead of NaCl, both the water-soluble proteins and myofibrillar proteins were subjected to SDS-PAGE analysis with 12% polyacrylamide gels and stained with Coomassie brilliant blue R-250, following the method of Jiang et al. [18].

### 2.12. Measurement of Carbonyl Content

The carbonyl content of the myofibrillar protein was determined by the method of Chen et al. [19] and expressed as nmol∙mg^−1^ protein. In total, 1 mL of 4 mg∙mL^−1^ myofibrillar proteins was mixed with 1 mL of 10 mM 2,4-dinitrophenylhydrazine (DNPH). Then, the solution was vortexed and added in 20% trichloroacetic acid (TCA) every 10 min in the dark conditions. After centrifugation at 8000 rpm for 5 min, the precipitate was washed 3 times with a mixture of absolute ethanol and ethyl acetate (1:1 *v*/*v*). Then, 3 mL of 6 M guanidine hydrochloride solution was added to dissolve the precipitate at 37 °C for 15 min. Then, the supernatant centrifugated at 8000 rpm for 5 min was collected, and the absorbance was measured at 370 nm. The carbonyl content was calculated using a molar extinction coefficient of 22,000 L∙mol^−1^∙cm^−1^.

(6) Carbonyl contentnmol/mg MP=V1×A1−A0V0×ε×d×ρ×1000

where $A_{1}\;$ is the absorbance of the sample at 370 nm,$\;A_{0}\;$ is the absorbance of the blank group at 370 nm,$\;V_{1}\;$ is the volume of the volume of guanidine hydrochloride solution,$\;V_{0}\;$ is the volume of protein sample solution to be measured, d is the colorimetric optical path, n is the dilution ratio, ε is the molar extinction coefficient 22,000 (L∙mol^−1^∙cm^−1^), ρ is protein concentration (mg∙mL^−1^).

### 2.13. Measurement of Total Sulfhydryl Groups and Active Sulfhydryl Groups Content

The total sulfhydryl groups and active sulfhydryl groups levels were determined according to the description of Zheng et al. [20] with a modification. As for total sulfhydryl group level, 1 mL MP solution (2.5 mg∙mL^−1^) with 9 mL 0.2 M Tris-HCl buffer C (containing 3 mM EDTA,8 M urea, 1%SDS, pH 8.0) was used. Then, 4.5 mL 4 mL of the above mixture was added to 0.5 mL 0.2 M Tris-HCl buffer D (containing 10 mM DTNB, pH 8.0). The suspension was incubated at 40 °C for 25 min and centrifuged at 10,000 r/min for 15 min. Buffer solution B was used as a blank control. The total sulfhydryl level was calculated by measuring the absorbance at 412 nm, and the molar absorbance extinction coefficient was 13,600 m^−1^ cm^−1^. A mixture of 4.5 mL of the above Tris-HCl buffer B and 0.5 mL of 0.1% 5, 5′-dithiol-bis (2-nitro-bis) benzoic acid was used as a blank control group. The determination method of active sulfhydryl groups level is the same as that of total sulfhydryl groups level, except that urea is not added to buffer solution. The level of the SH groups content was calculated using the following equation:

(7) $$SH\left(\mathrm{nmol}/\mathrm{mg}\;\mathrm{MP}\right)=\left(A\times n\right)/{\left(\varepsilon \times \rho \right)}^{\times 10^{6}}$$

where A is the absorbance of the sample at 412 nm, n is the dilution ratio, ε is the molar extinction coefficient 13,600 (L∙mol^−1^∙cm^−1^), ρ is protein concentration (mg∙mL^−1^).

### 2.14. Measurement of Proteins Surface Hydrophobicity

The surface hydrophobicity analysis of myofibrillar protein was determined using the BPB (bromophenol blue) as the indicator [21], and it followed the procedure with slight modification [22]. The myofibrillar proteins were dissolved in 10 mM phosphate buffer (containing 0.6 M NaCl, pH 6.0) and diluted to 2 mg∙mL^−1^. Each diluted myofibrillar solution (2 mL) was thoroughly vortexed for 10 min in 200 μL 1 mg∙mL^−1^ BPB solutions. Then, 25 mL 20 mM phosphate buffer B (containing 0.6 M NaCl, pH 7.0) was vortexed for 10 min in 200 μL BPB solutions (1 mg∙mL^−1^) using the same method, and measured as a blank group. The diluted supernatant was centrifuged at 5000 r∙min^−1^ for 15 min 10 times and the absorbance was measured at 595 nm. The absorbance at a wavelength of 340 nm was determined by a UV-1800PC spectrophotometer (Shanghai Mapada Instrument Co., Ltd., Shanghai, China).

(8) bound BPB contentμg=A0−A1A0×200 μg

where $A_{0}$ is the absorbance of blank group at 595 nm, $A_{1}$ is absorbance of sample at 595 nm.

### 2.15. Measurement of Secondary Structure Changes in Myofibrillar Protein

Half a gram of myofibrillar protein freeze-dried for 48 h was placed on the surface of the attenuation total reflection crystal. Fourier transform infrared spectroscopy was collected by a FTIR spectrometer (PerkinElmer, Seer Green, UK) to investigate the secondary structure conformation of myofibrillar proteins in samples. Omnic spectral software (Thermo Fisher Scientific Inc., Waltham, MA, USA) was used to analyze the original spectral data to obtain a quantitative estimation of secondary structure. 

### 2.16. Statistical Analysis

All experiments were conducted in triplicate. The obtained data were plotted onto graphs using Origin^®®^2018 (Origin Lab Corp., Northampton, MA, USA). One-way analysis of variance (ANOVA) was used to analyze the physiochemical parameters, performed using software SPSS^®®^26.0 (Chicago, IL, USA). All results were conducted as mean ± standard deviation (SD). Statistical significance was set at *p* < 0.05. The LSD procedure was used to compare different replacement proportions of NaCl with KCl. Pearson’s correlation between protein properties and quality attributes of lightly salted tilapias fillets with KCl addition was determined by SPSS^®®^26.0.

## 3. Results and Discussion

### 3.1. Changes in pH with Different KCl Replacements of Lightly Salted Tilapias Fillets

**Figure 1:**
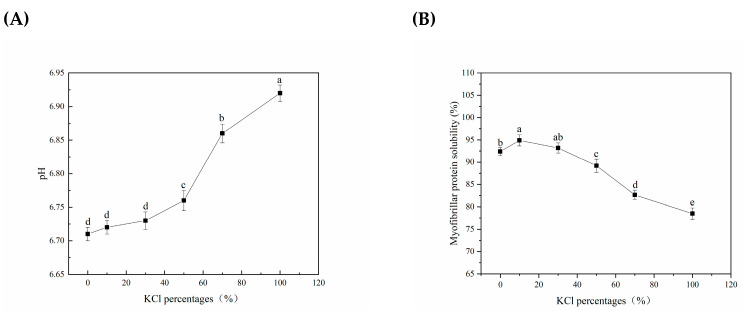
Change in pH and myofibrillar protein solubility of salted tilapia fillets as affected by different KCl replacements. (**A**) pH; (**B**) myofibrillar protein solubility. Values with different lowercase letters are significantly different at *p* < 0.05.

As shown in Figure 1A, KCl replacements could significantly increase the pH in samples compared to samples salted with NaCl (*p* < 0.05). Similar results for the higher pH under the partial or complete substitution of KCl were observed by Hand et al. [23] and Song et al. [15], who found an increase in the pH of tissues with an increasing percentage of KCl. It is generally believed to be related to the binding ability of Na^+^, K^+^ to muscle protein, or the difference in membrane permeability ability of Na^+^ and K^+^. Song et al. [15] noted a possible explanation that salting with KCl might be more effective in promoting high ionic strength within tissue, compared to salting with NaCl, owing to rapid penetration, thereby effectively inhibiting glycolysis value with KCl treatment, which eventually in turn contributed to the slightly higher ultimate pH [24]. However, the infiltration rate of K^+^ was higher than that of Na^+^ [21]. The increase in pH might be ascribed to protein hydrolysis under the action of K^+^ [4].

### 3.2. Changes in Myofibrillar Protein Solubility with Different KCl Replacements of Lightly Salted Tilapias Fillets

Myofibrillar protein solubility refers to the ratio of the amount of protein in muscle that could enter the solution to the total amount of muscle protein under certain conditions. Figure 1B showed that the myofibrillar protein solubility significantly decreased with substitutions up to 50% of NaCl by KCl (*p <* 0.05). The discrepancy in myofibrillar protein solubility might be induced by weakening the protein–water interactions and strengthening the protein–protein interactions [25]. This directly alters water’s hydrogen-bonding network at the proximity of the surfaces, and water distribution and state around the myofibrillar protein molecule [26], as supported by the carbonyl group content. 

### 3.3. Oxidative Stability with Different KCl Replacements of Lightly Salted Tilapias Fillets

**Figure 2:**
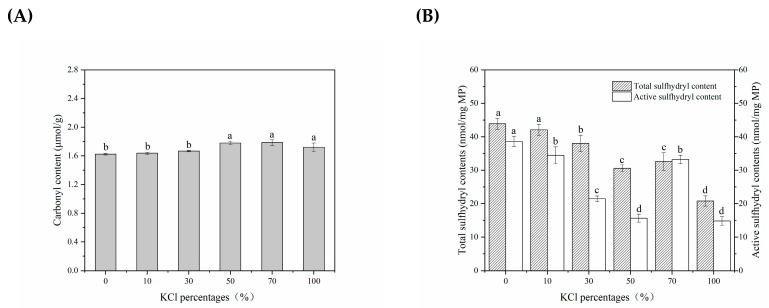
Change in oxidative stability of salted tilapia fillets as affected by different KCl replacements. (**A**) carbonyl content; (**B**) sulfhydryl group content. Values with different lowercase letters are significantly different at *p* < 0.05.

The oxidative stability of lightly salted tilapias fillets as affected by KCl replacements was investigated in terms of the carbonyl group content and sulfhydryl group content (see Figure 2). Carbonylation, as the most significant chemical modification of protein oxidation, was used to characterize the degree of protein oxidation. The presence of KCl replacements (50~100% KCl) favored a higher content of carbonyl groups (*p <* 0.05) in comparison to that with 100% NaCl, as shown in Figure 2A. These values increased to 1.79 nmol∙mg^−1^ (70% of KCl) and 1.72 nmol∙mg^−1^ (100% of KCl), which suggested that KCl replacements significantly promoted protein oxidation. Similar results were also obtained, showing that the chloride salts mixtures can better facilitate protein oxidation compared to the single NaCl at the same ionic strength. A similar result was also obtained by Zheng, Han, Ge, Zhao, and Sun [20]. Obviously, enhancing the electrostatic repulsion via Cl^−^ binding to actomyosin had been considered as a secondary cause in this study. This further confirmed that cation specificity was the key factor affecting protein properties in KCl replacements. The carbonyl group content might be correlated with protein degradation, but it also strongly depended on the endogenous enzymes surrounding the groups. It has also been reported that the K^+^ can facilitate more slack protein structures, further accelerating the oxidation of muscle protein [27]. Some water-soluble cellular substances were released from the sample as affected by KCl replacements, which might include antioxidant enzymes and other antioxidants, and this thus affected the oxidative stability of the sample [18].

The changes in active sulfhydryl groups and total sulfhydryl groups were measured, and the changes in disulfide bond content of myofibrillar protein processed with KCl were predicted. The total sulfhydryl group content included the active sulfhydryl groups on the surface (reactive sulfhydryl group) and the sulfhydryl groups located inside the protein [28]. In Figure 2B, the active and total sulfhydryl groups progressively decreased with KCl replacing NaCl (*p* < 0.05). The content of total sulfhydryl groups salted with 100% NaCl was 43.90 nmol∙mg^−1^, whereas the values decreased to 20.79 nmol∙mg^−1^ upon exposure to 100% KCl modification. The content of active sulfhydryl groups salted with 100% NaCl was 38.61 nmol∙mg^−1^, whereas the values decreased to 14.82 nmol∙mg^−1^ upon exposure to 100% KCl modification. The significant decline in total sulfhydryl groups (0~50% KCl) and active sulfhydryl groups (0~100% KCl) accompanied by increased KCl replacement meant that sulfhydryl groups were oxidized to intermolecular and intramolecular disulfide bonds, or further oxidized to products such as sulfonic acid. This agreed with findings reported by previous studies; all chloride salts raised MP’s disulfide bond level [20]. However, KCl partially replacing 70% NaCl led to a significant increase in the active sulfhydryl groups (*p* < 0.05); while, the total sulfhydryl groups content did increase significantly (*p* > 0.05), which could be attributed to the exposure of the buried sulfhydryl groups to the surface induced by KCl replacements. The rearrangement of protein molecules at this time kept the exposed sulfhydryl groups. Moreover, the presence of 70~100% KCl replacing NaCl showed a significant decrease in active and total sulfhydryl groups content (*p* < 0.05). In conclusion, KCl percentage within 50% accelerated the dehydration of protein in tissue; some side-chain residues were exposed to quickly form disulfide, hydrogen, hydrophobic bonds and so on, which were thought to be of crucial importance for changes in the aggregation and crossing links of proteins. 

### 3.4. Protein Structural Changes with Different KCl Replacements of Lightly Salted Tilapias Fillets

**Figure 3:**
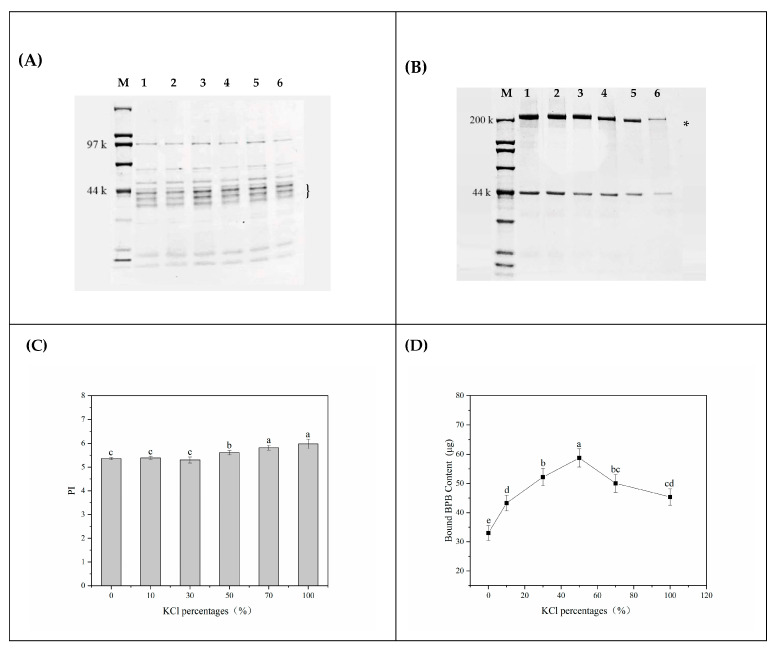
Change in protein structure stability of salted tilapia fillets as affected by different by-products. (**A**) SDS-PAGE profiles of water-soluble proteins; (**B**) SDS-PAGE profiles of myofibrillar proteins; (**C**) PI; (**D**) protein surface hydrophobicity. M: marker; 1, 2, 3, 4, 5, 6: myofibrillar proteins with different replacement proportions of NaCl with KCl (0%, 10%, 30%, 50%, 70%, 100%) in lightly salted tilapia fillets; The parentheses and asterisk indicate the band with a low molecular weight of approximately 15~40 kDa and a molecular weight of around 220 kDa in proteins fraction protein affected by KCl replacements. Values with different lowercase letters are significantly different at *p* < 0.05.

To clarify the changes in proteins of salted tilapias fillet with different KCl replacing NaCl, the SDS-PAGE profile of both water-soluble proteins and myofibrillar proteins are shown in Figure 3A,B. The band with a molecular weight of around 220 kDa (indicated by asterisk) in the myofibrillar proteins fraction was tentatively identified as myosin heavy chain (MHC). The amount of MHC became thin in these samples, as marked by an asterisk in the SDS-PAGE profiles, indicating myofibrils or actomyosin denaturation induced by KCl replacements. The discrepancy might be ascribed to the reduced activity of enzymes responsible for protein degradation to produce many small molecular proteins and further activation [29]. The solubilization of a water-soluble protein fraction corresponded with a low molecular weight of approximately 15~40 kDa (indicated by parentheses), tentatively identified as tropomyosin, troponin, myosin light chains, and their degradative placement of NaCl with KCl. Armenteros et al. [30] found that endoproteolytic enzymes such as cathepsins B and B + L appeared to be more active when more KCl and less NaCl were present. Therefore, we assume that KCl replacements probably influence this result by affecting the cathepsin activity, which was likely associated with the biochemical reactions in fillets.

Proteolysis index was an important index to indicate the degree of proteolysis, which was an important biochemical reaction in the processing of traditional salted fish products. The changes in the proteolytic index of lightly salted tilapia fillet treated with KCl in different proportions are shown in Figure 3C. The significant proteolysis of samples was accompanied by the replacement up to 50% of NaCl with KCl (*p* < 0.05). Upon exposure to K^+^ with high hydration capacity, proteolysis was induced through hydration layer reinforcement on the protein surface. This might suggest that K^+^, at a certain level, promoted the unfolding of protein conformation by exposing more sensitive amino acid side chains or specific amino acid specific binding sites to the protein-protein interface, which increased the accessibility of amino acids comprising hydrocarbon side chain groups in the hydrophobic inner core. Meanwhile, certain sensitive amino acid residues, such as proline, aspartic acid and glutamine, were oxidized, which could ultimately lead to protein degradation [31].

Protein secondary structure prediction was a fundamental and an important component in the study of protein structure and function. The relative content of the secondary structure content for myofibrillar protein was calculated in Table 1, including α-helix, β-turn, β-sheet, and random coil. α-helix, as the intramolecular ordered arrangement constructed by intramolecular hydrogen-bonding interactions, was the most stable structure of protein secondary structure. β-sheet was assigned to intermolecular β-sheets structures relating to the protein aggregation and antiparallel β-sheets structures. As shown in Table 1, with the increase in KCl substitution percentage, the relative content of α-helix and β-turn in the secondary structure of myofibrillar protein decreased (*p* < 0.05), whereas the relative content of β-sheet and random coil increased (*p* < 0.05). The relative α-helix content decreased from 22.74% (100% NaCl) to 19.37% (100% KCl), whereas the relative β-sheet content increased from 28.14% (100% NaCl) to 33.69% (100% KCl). These results reveal that with the increased KCl addition, α⁃helix transformed into β-sheet and random coil, strengthening the force to maintain the stability of secondary structure [32]. This condition indicates that protein structural change shifted from the proteins inside to the surface between molecules, accompanied by a greater degree of interaction between protein molecules, which made samples maintain a more stable structure. Additionally, with an enhanced KCl substitution percentage, a decreased relative content of α⁃ helix was observed. This could be due to the fact that the KCl substitution could promote the unfolding of protein molecules, as well as the extension of the molecular chain. The unfolding of molecular structure may promote the exposure of internal hydrophobic groups, as well as the generation of disulfide bonds. These results are basically consistent with the above determination of texture characteristics and sulfhydryl content.

Surface hydrophobicity impacted the tertiary structure of protein [33], as an important indicator of evaluating the construction stability of proteins and measuring the degree of protein denaturation. Protein surface hydrophobicity strongly depends on the type and concentrations of hydrophobic amino acids on the surface of protein molecules. According to Figure 3D, KCl substitution increased surface hydrophobicity as indicated by the combined BPB amount (*p* < 0.05), demonstrating the more exposed hydrophobic amino acid residues processed in KCl. In line with the result of carbonyl content, this might be due to the changes in protein secondary structure [34] by protein oxidation, contributed by the exposure of a large number of relatively dense hydrophobic regions originally embedded in myosin light chain (MLC) [22]. As the KCl substitution percentage further increases (70~100% KCl), the decreasing tendency of surface hydrophobicity contrary to the trend of protein oxidation also contributed a possibility to this hypothesis of cation specificity. It was also documented that at the same valency, Na^+^ with higher hydration radius stabilized the system more than K^+^ under high salt concentration conditions [7]. Along with the increased proportion of K^+^, the hydration stability is weakened. According to the stabilization mechanism on the hydrophilic nature of the interface reported by Molina-Bolivar, Galisteo-Gonzalez, and Hidalgo-Alvarez [12], proteins absorbed by K^+^ tend to change protein conformation by exposing more hydrophobic sites to the protein–water inter surface, oriented toward the hydrophobic polymer surface. Therefore, the difference in hydration ability was speculated to be the critical reason for changing the hydration properties of protein, which needs to be investigated in the further study of KCl replacements.

### 3.5. Quality Changes with Different KCl Replacements of Lightly Salted Tilapias Fillets

Quality properties of lightly salted tilapias with different KCl replacements were evaluated in terms of moisture content, yield, cooking loss, centrifuging loss, springiness, cohesiveness, lightness (L*), redness (a*), yellowness (b*), and sensory evaluation. 

**Figure 4:**
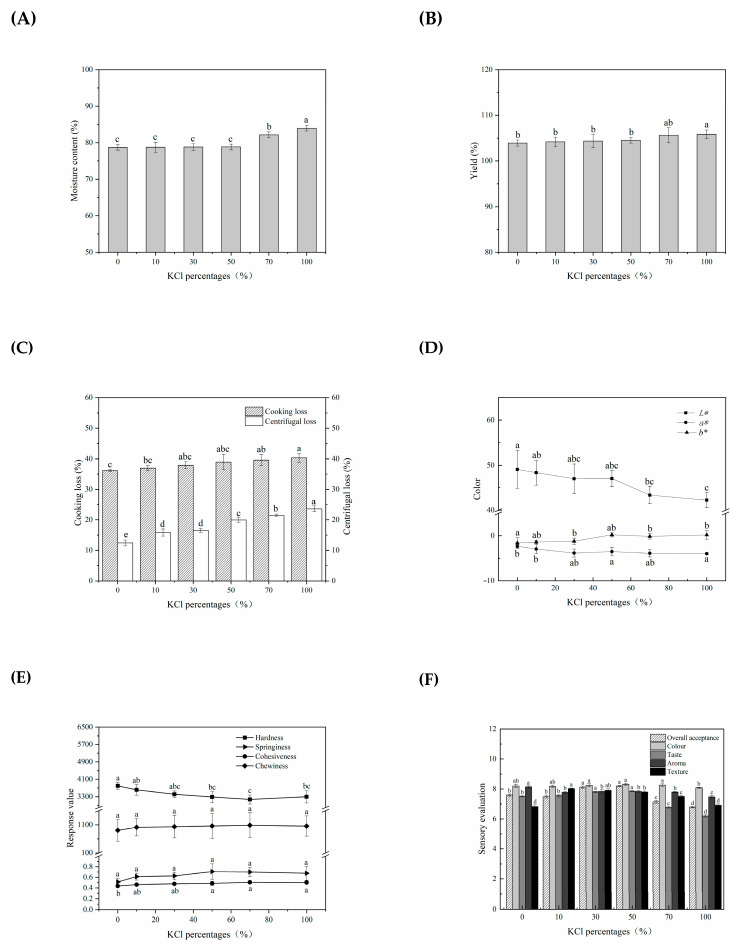
Change in quality properties of salted tilapia fillets as affected by different replacement of NaCl with KCl. (**A**) Moisture content; (**B**) yield; (**C**) water-holding capacity; (**D**) color; (**E**) texture profile; (**F**) sensory evaluation. Values with different lowercase letters are significantly different at *p* < 0.05.

The moisture content with different replacement percentages of NaCl by KCl was shown in Figure 4A. In this work, KCl-treated samples within 50% replacement proportion showed no significant difference in the moisture content with the control (*p* > 0.05); whereas, the moisture content significantly increased with the further increase in KCl percentage (*p* < 0.05). This situation might be due to the fact that with the well-off diffusion and osmosis under KCl replacements, there was decreased water loss flowing out of the sample. KCl replacements resulted in a higher salting efficiency, which was manifested as an increase in moisture content based on the binding ability of cations to proteins: Na^+^ > K^+^ [7]. In this work, adding K^+^ promoted the oxidative hydrolysis of protein and caused the expansion of myofibrils filament lattice and solubilization of myofibrillar proteins, which ultimately facilitated the moisture uptake during salting. As shown in Figure 3B, the results of the yield were consistent with the moisture content change in tissue along with increased partial replacement of NaCl by KCl, i.e., constant and increasing significantly. Replacement of NaCl with KCl had considerably increased the final yield beyond 70% of KCl, which might be the result of increased moisture content in tissue (*p* < 0.05). 

Cooking loss and centrifugal loss were analyzed, and the effect of KCl replacements in different proportions on the water-holding capacity (WHC) is demonstrated in Figure 4C. Higher centrifugal loss was found in samples with the substitution up to 50% of NaCl with KCl (*p* < 0.05), which was consistent with the previous observation [15,34]. The centrifugal loss (23.66%) at 100% of KCl was significantly higher than the 100% of NaCl (12.43%). A significant increase in the cooking loss with KCl replacements was observed compared to samples with NaCl (*p* < 0.05). The cooking loss (40.35%) at 100% KCl substitution proportion with KCl replacements was significantly (*p* < 0.05) higher than for those salted with NaCl in control (36.18%). Contradicting both the conclusion that the cooking loss was affected by ionic strength but not by salt type [7,15,34], and the speculation that Cl^−^ rather than Na^+^ acted as the protein surface charge [14], these results showed a significant effect using KCl replacements at both ionic strengths and ionic concentration 1.0256. This further suggested that cationic specificity rather than ion concentration and ion strength might have a major impact on WHC. Therefore, KCl replacements (50–70% KCl) caused the change in WHC of salted fillets, which might be ascribed to the higher moisture.

Color was a crucial factor affecting consumers’ desire to consume fish and fish products. It was also an important physical evaluation index of the sensory quality and a reference index of protein properties. As shown in Figure 4D, KCl replacing NaCl showed a significant decrease in the L* and a* values (*p* < 0.05) and significant increase in the b* value (*p* < 0.05). The decrease in the a* value might be related to the redox state of myoglobin [35], while the increase in the b* value might be associated with the degree of lipid oxidation, which proves that KCl could significantly affect the degree of lipid oxidation and lipolysis activity of samples [36]. The change of L* might be related to the changes in protein construction. Changes in protein structure and aggregation between myofibrils influenced the light reflection characteristics and yellowness of samples. Structural changes or degradation of protein were often accompanied by changes in microstructure, such as z-line fragmentation and loss of A-band and M-line [37]. These changes might increase the transverse scattering of the light beams, thus playing a role in the color of samples. These results were in agreement with those obtained by Tahergorabi et al. (2012) who found that the L*and a* values of surimi gels significantly decreased with the substitution of NaCl with KCl.

A departure from expected texture characteristics, mainly the loss of juiciness and harsh mouthfeel, has been a question that has received much more concern with KCl substitution, making it less preferred by consumers [38]. Figure 4E summarized the texture characteristics of tilapia fillets co-salted with KCl and NaCl in varying proportions. As the KCl substitution proportions increased, the hardness stayed constant at first (*p* > 0.05) and then significantly increased (*p* < 0.05), reaching the maximum value (3297.89 g) at a substitution proportion of 100% KCl. The increased hardness when salted with KCl might be attributed to the lower hydration of the K^+^ ion than that of the Na^+^ ion acting on the interaction with muscle proteins [39], the type and amount of extracted proteins, as well as moisture loss. It was suggested that KCl replacements could be an alternative means to alleviate the problem of texture softening in salted fillets, and, in turn, provide the advantage of juiciness and tenderness. The cohesiveness increased significantly when the KCl percentage reached 50% (*p* < 0.05), which might be related to the increase in protein–protein interaction. However, KCl replacements showed no significant difference in chewiness and springiness compared with NaCl-treated fillets (*p* > 0.05). 

Sensory characteristics of KCl-treated tilapia fillets with different KCl percentage were evaluated from the five aspects of color, texture, taste, aroma, and overall acceptance. As shown in Figure 4F, the texture score had no significant difference between the KCl-treated samples and the control, but the lower score was shown in higher KCl content (50~70% KCl) than lower KCl content (10~30% KCl). This may be due to the relatively higher moisture content. There was no significant difference in color between the KCl treatment and the control (*p* > 0.05), which suggested that KCl-treated samples were not enough to receive color recognition. KCl replacing NaCl led to a significant decrease in the aroma (*p* < 0.05). The intensities of taste showed apparent differences among these groups (*p* > 0.05). The highest values were shown in 50% KCl (*p* < 0.05), and low values were found with further KCl substitution (70% of KCl and 100% of KCl), respectively. This result demonstrated that KCl percentage within 50% showed the most improvement in the sensory quality of KCl-treated samples, which is similar to these previous reports [40,41].

### 3.6. Relationship between Protein Properties and Quality Attributes with Different KCl Replacements of Lightly Salted Tilapias Fillets

**Figure 5:**
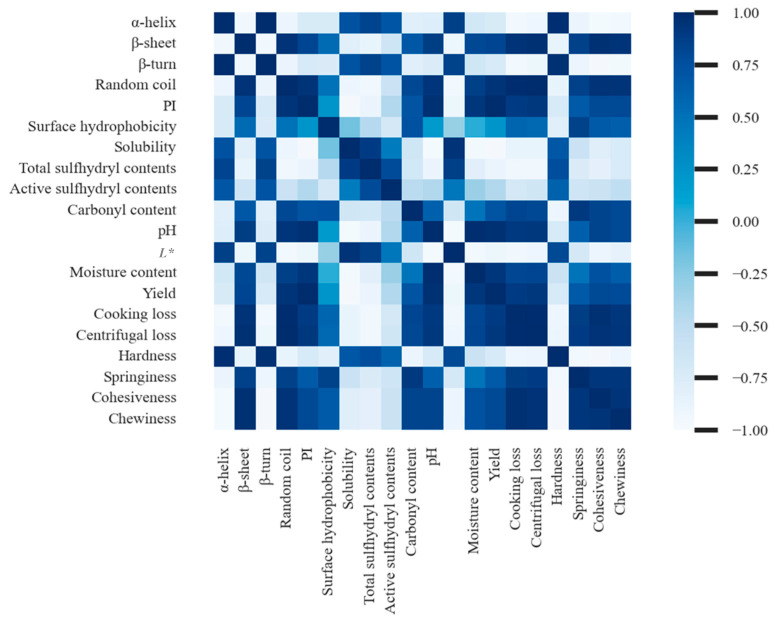
Pearson’s correlation analysis and levels of significance for correlations between protein properties and quality attributes with different KCl replacements of salted tilapia fillets as affected by different replacement of NaCl with KCl.

As shown in Figure 5, there were significant relationships among the moisture content, yield, and protein solubility in tissue, along with increased partial replacement of NaCl by KCl. WHC were correlated with protein construction and myofibrillar proteins solubilization, as indicated by α-helix, β-turn, β-sheet, random coil, proteolysis index, and protein surface hydrophobicity, which suggested that the myofibrillar protein network structure became loose and disordered to make moisture easier to evaporate or lose during cooking or centrifuging with KCl replacements. Correlation analysis also showed that there was no significant correlation between proteolysis and hardness, which showed that partial replacement of NaCl by KCl accelerated proteolysis without affecting the texture of lightly salted tilapia fillets in this study. In addition, protein oxidation with a high percentage of KCl facilitated the formation of carbonyl groups and disulfide bonds, thereby inducing the disassembly of myofibrils and the formation of intermolecular interactions. This change in protein structure increased the cohesiveness and hardness in tissue. The structure of proteins was also the main factor to affect the brightness, which could alter the light-scattering properties. In general, the sensory quality was influenced by the proteolysis and myofibrillar protein structure of the salted tilapias fillets.

## 4. Conclusions

At a moderate KCl replacement (within 50% of KCl), samples had a stable moisture content, water-holding capability, as well as yield (*p* > 0.05), corresponding to 100% NaCl. These results indicated that the replacement percentage up to 50% of NaCl with KCl is adequate to obtain an acceptable fish product without affecting its color and sensory characteristics. KCl replacing NaCl could show certain effects on the textural properties of salted tilapias fillets to a certain extent. Further KCl replacement improved the springiness and cohesiveness of tilapia fillets, and the hardness increased slightly with NaCl-salted fillets, protecting against texture softening problems to a certain extent. The dimmed brightness of fillets was not seen to such an extent as to receive sensory recognition. Muscle tissue could absorb moisture based on the superior penetration efficiency by K^+^, but samples had a weak water-holding capacity when subject to centrifugation and cooking. Excessive KCl substitution at a high proportion (more than 70%) was detrimental to quality properties, manifested as lower WHC, dimmed color, and sensory deterioration by increased hardness and extreme bitterness, leading to the worst sensory acceptability. This might be ascribed to the insolubilization and weakened stability of myofibrillar proteins based on the difference in binding order and hydration capability between K^+^ and Na^+^. Protein tended to change the structure, represented by the unfolding of the myofibrillar protein, and increased surface hydrophilic point. Additionally, it strengthened the internal protein–protein interaction, resulting in structural reinforcement and decreased water-holding capacity. Immoderate protein hydrolysis and aggravated oxidation of myofibrillar protein caused discoloration and taste deterioration. The findings can provide great theoretical and applicable value to the process and application of low-sodium seafood products. However, further study is needed to reveal the specific relationship with the quality changes in fish, especially regarding the microstructure and the underlying mechanisms. 

## Figures and Tables

**Table 1:** Change in MP secondary structure relative content of salted tilapia fillets as affected by partial replacement of NaCl with KCl.

KCl Percentages (%)	α-Helix (%)	β-Sheet (%)	β-Turn (%)	Random Coil (%)
0%	22.74 ± 0.29 ^a^	28.14 ± 0.13 ^d^	22.74 ± 0.29 ^a^	25.48 ± 1.15 ^c^
10%	21.75 ± 0.94 ^a^	30.52 ± 1.12 ^c^	21.75 ± 0.74 ^a^	26.81 ± 0.35 ^cd^
30%	20.22 ± 0.85 ^b^	31.08 ± 0.18 ^c^	20.22 ± 0.55 ^b^	27.02 ± 0.88 ^c^
50%	20.14 ± 0.80 ^b^	31.35 ± 0.31 ^c^	20.14 ± 0.6 ^b^	28.64 ± 0.52 ^b^
70%	19.45 ± 1.18 ^b^	32.58 ± 0.64 ^b^	19.67 ± 0.38 ^b^	29.83 ± 0.92 ^ab^
100%	19.37 ± 0.21 ^b^	33.69 ± 0.56 ^a^	19.45 ± 1.11 ^b^	30.61 ± 0.42 ^a^

Data are shown as means ± standard deviations (*n* = 3). Data in the same row with different lowercase letters are significantly different (*p* < 0.05).

## Data Availability

The data used to support the findings of this study can be made available by the corresponding author upon request.
